# Magnetic Resonance Imaging Features for Distinguishing High-Grade From Low-Grade Soft Tissue Sarcoma: A Systematic Review and Meta-Analysis

**DOI:** 10.7759/cureus.72784

**Published:** 2024-10-31

**Authors:** Wilhelmina N Hauwanga, Billy McBenedict, Kang Suen Goh, Ryan Chun Chien Yau, Anusha Thomas, Berley Alphonse, Yusuf A Ahmed, Walaa H Yusuf, Jeshua N Devan, Hind A Alsiddig, Abdelwahab Ahmed, Bruno Lima Pessôa

**Affiliations:** 1 Cardiology, Federal University of the State of Rio de Janeiro, Rio de Janeiro, BRA; 2 Neurosurgery, Fluminense Federal University, Niterói, BRA; 3 Internal Medicine, Monash University Malaysia, Johor Bahru, MYS; 4 Neurology, Christian Medical College, Ludhiana, Ludhiana, IND; 5 Internal Medicine, University Notre Dame of Haiti, Port-au-Prince, HTI; 6 General Surgery, Dammam Medical Complex, Dammam, SAU; 7 Surgery, Asian Institute of Medicine, Science and Technology (AIMST University), Kedah, MYS; 8 Radiology, Khartoum Hospital, Khartoum, SDN; 9 Internal Medicine, Aswan University Hospital, Aswan, EGY

**Keywords:** heterogeneity, magnetic resonance imaging (mri), necrotic signal, peritumoral enhancement, soft tissue sarcoma, tumor grade

## Abstract

Soft tissue sarcomas are malignant tumors characterized by heterogeneity and are associated with a high mortality rate. Histopathological grading is considered a pivotal factor in prognostication and treatment planning. While core needle biopsy exhibits high accuracy in determining tumor histology, it fails in some cases, potentially misclassifying high-grade tumors as low-grade. Magnetic resonance imaging (MRI) has been evaluated as an adjunctive tool for predicting histopathological tumor grade. This systematic review and meta-analysis evaluated MRI features capable of distinguishing high-grade from low-grade tumors in patients with soft tissue sarcoma. A literature search was carried out in PubMed, Embase, and Cochrane Central in May 2024. The following features were evaluated for both low-grade and high-grade tumors: tumor size, heterogeneity on T2, presence of necrotic areas, margin definition on T1, and post-contrast peritumoral enhancement. Statistical analysis was conducted using the OpenMeta[Analyst] software (Providence, RI: Brown University), applying random effects models for pooled analyses with a 95% confidence interval (CI) based on the inverse variance method. A total of four studies, involving 343 patients categorized by tumor grade (high-grade or low-grade), who underwent MRI, were included in the analysis. The meta-analysis found similar incidences of tumor sizes less than 5 cm in both high-grade and low-grade tumors (22.7%; 95% CI: 10.3-25% vs. 27%; 95% CI: 2.7-51.2%) and tumor sizes greater than 5 cm (71.3%; 95% CI: 64-78.6% vs. 52%; 95% CI: 23.6-80.5%). High-grade tumors showed a higher incidence of post-contrast peritumoral enhancement compared to low-grade tumors (66%; 95% CI: 43-89% vs. 26%; 95% CI: 4.6-47.4%) as well as heterogeneity on T2 greater than 50% (72.4%; 95% CI: 49.3-95.4% vs. 25.4%; 95% CI: 5.2-56%). Additionally, high-grade tumors had a lower incidence of the absence of necrotic signal compared to low-grade tumors (28.8%; 95% CI: 8.5-49.1% vs. 68%; 95% CI: 57.5-78.6%). Our findings suggest that post-contrast peritumoral enhancement, presence of necrotic areas, and heterogeneity on T2 greater than 50% are MRI features associated with high-grade tumors in soft tissue sarcoma. Tumor size, however, does not appear to be a reliable indicator for differentiating tumor grade.

## Introduction and background

Soft tissue sarcomas (STS) represent a diverse and complex group of malignancies arising from connective tissues, encompassing over 100 distinct histologic and molecular subtypes. Each subtype exhibits unique clinical behaviors and characteristics, contributing to the overall heterogeneity of STS [1]. A comprehensive understanding of the histopathology of STS necessitates an in-depth analysis of their microscopic features, immunophenotypic profiles, and molecular characteristics. This detailed examination is crucial for informing clinical decision-making and tailoring appropriate treatment strategies. The intricate interplay of these factors underscores the importance of a meticulous and nuanced approach to diagnosing and managing STS.

STS can affect individuals of all ages but shows a slightly higher incidence in males, with a male-to-female ratio of approximately 1.4:1 [2]. The median age at diagnosis for STS is 59 years. The incidence of STS follows a bimodal distribution, with notable peaks occurring in the fifth and eighth decades of life [2,3]. There are over 50 distinct histologic subtypes of STS. Among these, rhabdomyosarcoma is the most common subtype in children, while undifferentiated pleomorphic sarcoma predominates in adults [4]. STS most frequently develops in the extremities. Specifically, tumors in the upper extremities account for 12% of all STS cases, while those in the lower extremities represent 28% of cases. Within the extremities, the thigh is the most common site for STS, comprising 44% of all cases in this region [5].

STS can arise from various etiological factors, including non-sporadic causes. Epidemiological risk factors for STS include viral infections, such as Epstein-Barr virus, and exposure to radiation [6]. Additionally, specific genetic abnormalities can contribute to the development of STS. Germline mutations that lead to the inactivation of one allele of a tumor-suppressor gene are known to play a role. For example, mutations associated with peripheral nerve sheath tumors are linked to neurofibromatosis type I, which increases the risk of developing STS [6]. Despite these associations, the majority of STS cases occur sporadically [7].

Patients with STS often present to primary care with large, painless soft tissue masses [8]. Given the relatively rare occurrence of STS compared to benign soft tissue tumors, which are approximately 100 times more common, these masses are frequently misdiagnosed as benign conditions [9]. This diagnostic challenge can lead to delays in referral to specialized sarcoma centers, which in turn may result in significant morbidity or even mortality [10]. Early detection and accurate differentiation between high-grade and low-grade STS are crucial for effective treatment planning. High-grade lesions typically require initial treatment with radiotherapy or neoadjuvant chemotherapy before surgical resection [11]. The management of STS remains complex and challenging, as even with complete resection and negative margins, the disease is prone to recurrences and metastases, often resulting in severe outcomes [8].

Several imaging modalities are used to investigate STS. For superficial STS, ultrasound is commonly employed due to its accessibility and ability to assess the size and depth of the mass [12]. Ultrasound guidance is also crucial for performing core biopsies of these superficial lesions. However, for staging and evaluating malignancy, magnetic resonance imaging (MRI) and computed tomography (CT) are preferred. MRI is particularly valuable for its high-resolution imaging and superior soft tissue contrast, making it effective in detecting STS and assessing anatomical details, including surrounding edema, heterogeneous signals, and potential bone destruction [13,14]. MRI can also delineate neurovascular structures and muscular compartments affected by the tumor. CT is used when MRI is contraindicated or when better delineation of cortical destruction or microcalcification is needed, particularly if calcification is noted on an X-ray [13]. Additionally, CT is crucial for systemic staging to identify pulmonary metastases and is used for imaging guidance during core-needle biopsy sampling. Despite the utility of these imaging techniques, biopsy remains the gold standard for confirming an STS diagnosis [14].

Currently, MRI is considered the most effective imaging modality for STS due to its precise tumor localization capabilities and its ability to detail anatomical landmarks and vascularization relative to the tumor [15]. MRI is particularly adept at differentiating between high-grade and low-grade STS based on heterogeneous signal intensities, tumor size, and margins [16]. Research by Fernebro et al. [15] and Zhao et al. [17] indicates that high-grade sarcomas often present with larger size, higher heterogeneous signal intensity, poorly defined margins, and increased peritumoral signal intensity on T2-weighted images. Conversely, low-grade tumors are more frequently hyperintense on T1-weighted images [15,17]. Despite these findings, there is limited research focusing on the specific MRI characteristics that differentiate high-grade from low-grade STS. This paper aims to conduct a single-arm meta-analysis to assess MRI features that effectively distinguish between high-grade and low-grade tumors in STS patients, highlighting the critical role of early detection and diagnosis in preventing treatment delays.

## Review

Methods

Search Strategy

This systematic review was conducted based on the recommendations of the Cochrane Collaboration guidelines, and the Preferred Reporting Items for Systematic Reviews and Meta-Analyses (PRISMA) statement was followed. A systematic search was performed in May 2024 across the MEDLINE, Embase, and Cochrane CENTRAL databases using the following search strategy: ("magnetic resonance" OR "MR" OR "MRI") AND ("soft tissue sarcoma" OR "soft-tissue sarcomas" OR "soft tissue sarcomas") AND ("tumor grade" OR "tumor grading" OR "histological grade" OR "histologic grade"). Additionally, references of included studies and systematic reviews were screened to identify new studies. A pre-established protocol was used but it was not registered in a protocol database. All discrepancies were resolved by consensus between two authors.

Inclusion and Exclusion Criteria

We included studies that met the selection criteria based on the pre-established PICOTT framework. The inclusion criteria required that studies involve individuals diagnosed with STS who underwent MRI and provided MRI characteristics for both low-grade and high-grade tumors. No restrictions were applied regarding the country of origin or language of the studies. The MRI characteristics analyzed in these studies included tumor size, signal heterogeneity, presence of necrotic areas, margin definition, and post-contrast peritumoral enhancement. These factors were chosen to provide a comprehensive evaluation of how MRI can differentiate between low-grade and high-grade STS.

Data Synthesis and Statistical Methods

For this study, a single-arm meta-analysis was selected as the analytical approach, conducted separately for data from low-grade and high-grade STS groups. The aim was to identify and compare the prevalence of specific MRI characteristics associated with each classification of STS. Rates of these characteristics were compared between the low-grade and high-grade groups to determine which features are most indicative of tumor grade. Data from the studies were pooled using the inverse variance method, employing a random-effects model to account for variability among studies. Outcomes and their confidence intervals were calculated, and forest plots were generated with OpenMeta[Analyst] software (Providence, RI: Brown University) to visually represent the results. The degree of heterogeneity among the studies was assessed using the I² test, providing insight into the consistency of the findings across different studies.

Results****

**Figure 1:**
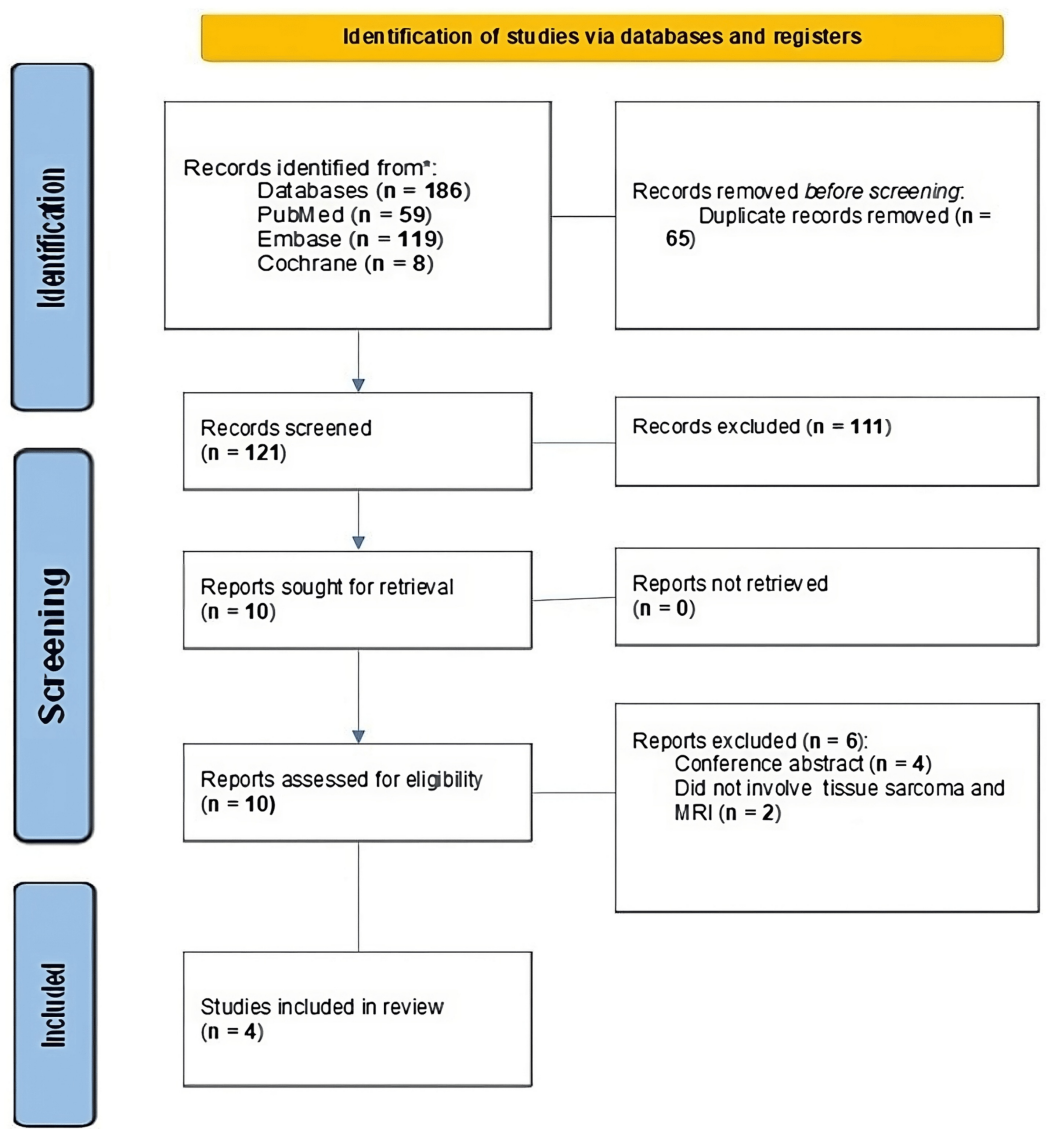
Preferred Reporting Items for Systematic Reviews and Meta-Analyses (PRISMA) flow diagram

Based on the comprehensive search strategy applied, a total of 186 studies were initially identified across the databases. After removing duplicates and conducting a preliminary screening of titles and abstracts, 10 studies were selected for full-text review. Following this detailed evaluation, four studies were ultimately included in the analysis. These studies collectively involved 343 patients and specifically reported on MRI characteristics distinguishing between low-grade and high-grade soft STS, as illustrated in Figure 1. The studies included in this review were observational in nature and originated from China, Brazil, and France. The characteristics of these studies, including their methodologies, patient demographics, and key findings, are summarized in Table 1. This selection process ensures that the analysis is based on robust and relevant data, providing valuable insights into the MRI features associated with different grades of STS.

**Table 1:** Baseline characteristics of included studies

Author	Country	Study design	Inclusion period	Number of patients	Number of males
Zhao et al. [17]	China	Observational	2010-2013	95	47
Marques et al. [18]	Brazil	Observational	2015-2022	68	38
Li et al. [19]	China	Observational	2016-2019	50	22
Crombé et al. [20]	France	Observational	2008-2015	130	77

The analysis revealed that the presence of heterogeneous signal intensity on T2-weighted MRI images when observed in more than 50% of the tumors was a highly effective indicator for distinguishing high-grade tumors from low-grade tumors (72.4% vs. 25.4%). In contrast, STS lacking necrotic areas were more frequently associated with low-grade tumors compared to high-grade tumors (68% vs. 28.8%). Additionally, post-contrast peritumoral enhancement was significantly more prevalent in high-grade tumors than in low-grade tumors (66% vs. 26%). Margin definition on T1-weighted MRI images, with well-defined margins, also proved to be a valuable feature for differentiating between low-grade and high-grade tumors. Detailed incidence rates for each MRI characteristic, along with their confidence intervals, are presented in Table 2.

**Table 2:** Meta-analysis of MRI characteristics in high-grade and low-grade soft tissue sarcomas

	High-grade		Low-grade	
MRI features	Number of study arms	Estimate (95% CI)	Number of study arms	Estimate (95% CI)
Size tumor < 5 cm	4	22.7% (10.3-35%)	4	27% (2.7-51.2%)
Size tumor ≥ 5 cm	4	71.3% (64-78.6%)	4	52% (23.6-80.5%)
Post-contrast peritumoral enhancement	4	66% (43-89%)	4	26% (4.6-47.4%)
Heterogeneity sign on T2‐weighted sequence > 50%	2	72.4% (49.3-95.4%)	2	25.4% (5.2-56%)
Heterogeneity sign on T2‐weighted sequence < 50%	2	23.9% (10.9-36.8%)	2	35.5% (8.8-79.9%)
No area with necrotic signal	2	28.8% (8.5-49.1%)	2	68% (57.5-78.6%)
< 50% with necrotic signal	2	40.4% (29.9-50.9%)	2	22.4% (13-31.8%)
> 50% with necrotic signal	2	31.7% (0.6-62.8%)	2	8.8% (0.7-16.9%)
Margin definitions at T1-weighted imaging (well-defined)	2	30.1% (22.3-38%)	2	56.6% (38-75.3%)

Regarding tumor size, MRI showed a slight difference for tumors smaller than 5 cm between high-grade and low-grade classifications (22.7% vs. 27%), while for tumors larger than 5 cm, a more notable difference was observed (71.3% vs. 52%). This pattern was also observed for heterogeneity signals on T2-weighted images with less than 50% heterogeneity (23.9% vs. 35.5%) and for the presence of necrotic areas with less than 50% involvement (40.4% vs. 22.4%), as detailed in Table 2.

Discussion

The prognostic value of MRI features in differentiating high-grade from low-grade STS is strongly supported by this study's findings. The study confirms that specific MRI characteristics, such as peritumoral enhancement, necrosis, and heterogeneous signal intensities on T2-weighted imaging, are significantly associated with high-grade STS. These features offer valuable insights into the aggressiveness of the tumor, aiding in clinical decision-making. A notable finding is that tumors measuring 5 cm or larger are more likely to be high-grade, with 71.3% of such tumors categorized as high-grade compared to 52% for low-grade tumors. This reinforces the utility of MRI in evaluating the aggressive nature and prognosis of STS, facilitating more precise diagnostic and therapeutic strategies. These results align with previous studies that have highlighted tumor size as a critical prognostic factor for STS [17]. Additionally, peritumoral enhancement emerged as a strong indicator of high-grade tumors, with 66% of high-grade STS showing this feature compared to only 26% of low-grade STS. This finding is consistent with research by Yoo et al. [21], which also identified peritumoral enhancement as a significant predictor of high-grade sarcomas. Overall, these results underscore the potential of MRI to enhance the accuracy of STS diagnosis and treatment planning by identifying features indicative of tumor grade and aggressiveness.

Crombé et al. [20] conducted a study investigating MRI features that can distinguish high-grade from low-grade STS and their correlation with patient outcomes. The study included 130 patients, with 55.4% (72 patients) classified as having grade III STS. The researchers found that several MRI characteristics were significantly associated with high-grade STS. Specifically, peritumoral enhancement was strongly correlated with high-grade tumors, showing an odds ratio (OR) of 3.4 (P = 0.003). The presence of necrosis within the tumor had an OR of 2.4 (P = 0.03) and heterogeneous signal intensities exceeding 50% on T2-weighted imaging had an OR of 2.3 (P = 0.04).

These results highlight the effectiveness of MRI in assessing the aggressive nature and prognosis of STS, thereby supporting more accurate diagnostic and therapeutic strategies. The study also demonstrated that the combination of two or three MRI features such as ´tumor necrosis, signal intensity heterogeneity, and peritumoral enhancement´ achieved a sensitivity of 75% and an accuracy of 70.2% in predicting grade III STS. This underscores the utility of MRI in evaluating tumor grade and guiding treatment decisions. Additionally, Zhao et al. [17] similarly identified peritumoral edema, margin definition, and enhancement as indicators of high-grade tumors, further validating the role of MRI in complementing histologic grading and improving patient outcomes.

The presence of heterogeneous signal intensities greater than or equal to 50% on T2-weighted imaging was found to be an independent predictor of high-grade STS, with an odds ratio of 2.3. This is in line with the hypothesis that tumor heterogeneity reflects intrinsic tumor aggressiveness and correlates with higher histologic grades [22]. Moreover, the presence of necrotic areas within the tumor was significantly associated with high-grade STS, supporting the notion that necrosis is a marker of poor prognosis and higher tumor grade [23]. Interestingly, the study found that the absence of necrotic signal is more indicative of low-grade STS (68%), while the presence of less than 50% necrotic signal within the tumor volume is more associated with high-grade STS (40.4%). This underscores the importance of carefully assessing the extent of necrosis in MRI evaluations to improve the accuracy of tumor grading.

Well-defined margins on T1-weighted imaging were more frequently observed in low-grade STS (56.6%) compared to high-grade STS (30.1%), The findings of this study suggest that incorporating MRI features into the diagnostic and prognostic assessment of STS can enhance the accuracy of grading and provide complementary information to histologic analysis. This approach can potentially reduce the underestimation of tumor grade in biopsy samples, which is a common issue due to tumor heterogeneity. By identifying high-grade areas through MRI, radiologists can better guide biopsies and improve the initial grading accuracy. The study by Zhao et al. [17] found that tumor margin is an important indicator to predict the grade of tumor. A general concept was that if the tumor margin was partially or poorly defined on an MRI, it suggested signs of infiltration and aggressive tumor growth. In addition, the peripheral growth of the tumor has been proven to be an important prognosticate factor for metastasis or local recurrence of the STS [17]. This finding is aligned with the outcomes reported from the study by Marques et al. [18], wherein they discovered that the high‐intensity signal of peritumoral on T2‐weighted is significant with an odds ratio of 11.18. However, the results of the study by Li et al. [19] did not find a significant difference in the peritumoral enhancement between the high-grade STS and low-grade STS.

## Conclusions

The current meta-analysis revealed that post-contrast peritumoral enhancement, the area with a necrotic sign, and the sign of heterogeneity on T2 > 50% are MRI features that may be associated with a high-grade tumor in STS. Additionally, MRI results showed slight differences in classification for tumors smaller than 5 cm between high-grade and low-grade sarcomas, with more pronounced differences in larger tumors. These findings emphasize the importance of MRI features, including tumor size, in distinguishing between high-grade and low-grade STS. Overall, the study emphasizes the importance of MRI in the evaluation of STS and highlights the need for further research to refine these imaging markers and integrate them with molecular analyses for a more comprehensive assessment of tumor grade and prognosis. Future efforts should focus on developing standardized imaging protocols and exploring the potential of radiogenomics to enhance the predictive value of MRI features.

## References

[1] Weiss AR, Harrison DJ (2024). Soft tissue sarcomas in adolescents and young adults. J Clin Oncol.

[2] Howlader N, Noone A-M, Krapcho M (2018). SEER Cancer Statistics Review (CSR) 1975-2014. National Cancer Institute. Bethesda, MD.

[3] Bauer HC, Trovik CS, Alvegård TA (2001). Monitoring referral and treatment in soft tissue sarcoma: study based on 1,851 patients from the Scandinavian Sarcoma Group Register. Acta Orthop Scand.

[4] Dasgupta R, Fuchs J, Rodeberg D (2016). Rhabdomyosarcoma. Semin Pediatr Surg.

[5] Brennan MF, Antonescu CR, Moraco N, Singer S (2014). Lessons learned from the study of 10,000 patients with soft tissue sarcoma. Ann Surg.

[6] Kleinerman RA, Tucker MA, Abramson DH, Seddon JM, Tarone RE, Fraumeni JF Jr (2007). Risk of soft tissue sarcomas by individual subtype in survivors of hereditary retinoblastoma. J Natl Cancer Inst.

[7] Weskamp P, Ufton D, Drysch M (2022). Risk factors for occurrence and relapse of soft tissue sarcoma. Cancers (Basel).

[8] Morrison BA (2003). Soft tissue sarcomas of the extremities. Proc (Bayl Univ Med Cent).

[9] Casali PG, Blay JY (2010). Soft tissue sarcomas: ESMO clinical practice guidelines for diagnosis, treatment and follow-up. Ann Oncol.

[10] Ebrahimpour A, Chehrassan M, Sadighi M, Karimi A, Azizmohammad Looha M, Jafari Kafiabadi M (2022). Soft tissue sarcoma of extremities: descriptive epidemiological analysis according to national population-based study. Arch Bone Jt Surg.

[11] Boudabbous S, Hamard M, Saiji E, Gorican K, Poletti PA, Becker M, Neroladaki A (2022). What morphological MRI features enable differentiation of low-grade from high-grade soft tissue sarcoma?. BJR Open.

[12] Vodanovich DA, M Choong PF (2018). Soft-tissue sarcomas. Indian J Orthop.

[13] Choong PFM, Sim FH (2000). Tumours. Curr Orthop.

[14] Soper JR, Brown WE, Schatz JA Radiology of bone and soft tissue sarcomas. Cancer Forum.

[15] Fernebro J, Wiklund M, Jonsson K, Bendahl PO, Rydholm A, Nilbert M, Engellau J (2006). Focus on the tumour periphery in MRI evaluation of soft tissue sarcoma: infiltrative growth signifies poor prognosis. Sarcoma.

[16] Hong JH, Jee WH, Jung CK, Chung YG (2020). Tumor grade in soft-tissue sarcoma: prediction with magnetic resonance imaging texture analysis. Medicine (Baltimore).

[17] Zhao F, Ahlawat S, Farahani SJ, Weber KL, Montgomery EA, Carrino JA, Fayad LM (2014). Can MR imaging be used to predict tumor grade in soft-tissue sarcoma?. Radiology.

[18] Marques TM, Cerqueira WS, Neto JL (2024). Role of magnetic resonance imaging in the prediction of histological grade in soft tissue sarcomas. J Surg Oncol.

[19] Li X, Wang Q, Dou Y, Zhang Y, Tao J, Yang L, Wang S (2020). Soft tissue sarcoma: can dynamic contrast-enhanced (DCE) MRI be used to predict the histological grade?. Skeletal Radiol.

[20] Crombé A, Marcellin PJ, Buy X (2019). Soft-tissue sarcomas: assessment of MRI features correlating with histologic grade and patient outcome. Radiology.

[21] Yoo HJ, Hong SH, Kang Y (2014). MR imaging of myxofibrosarcoma and undifferentiated sarcoma with emphasis on tail sign; diagnostic and prognostic value. Eur Radiol.

[22] Corino VD, Montin E, Messina A, Casali PG, Gronchi A, Marchianò A, Mainardi LT (2018). Radiomic analysis of soft tissues sarcomas can distinguish intermediate from high-grade lesions. J Magn Reson Imaging.

[23] Monsky WL, Jin B, Molloy C (2012). Semi-automated volumetric quantification of tumor necrosis in soft tissue sarcoma using contrast-enhanced MRI. Anticancer Res.

